# Impact of Patients Decision Aids on Shared Decision‐Making and Patient Satisfaction Prior to Pelvic Floor Surgery

**DOI:** 10.1111/1471-0528.18103

**Published:** 2025-02-19

**Authors:** Ruth Athey, Roberta Bugeja, Georgina Jones, Swati Jha

**Affiliations:** ^1^ Sheffield Teaching Hospitals NHS Foundation Trust Sheffield UK; ^2^ Leeds Beckett University Leeds UK; ^3^ University of Sheffield Sheffield UK

**Keywords:** decision‐making, patient decision aid, PtDA, stress urinary incontinence, uterine prolapse, vault prolapse

## Abstract

**Objective:**

Evaluate usability and utility of National Institute for Clinical Excellence (NICE) Patient Decision Aid's (PtDA's) for pelvic floor surgery. PtDA's reviewed were uterine prolapse, vault prolapse and stress urinary incontinence (SUI).

**Design:**

Ten women given the PtDA's during routine clinical care were recruited from each cohort and underwent a semi‐structured interview with a clinical researcher.

**Setting:**

Urogynaecology outpatients in an NHS tertiary teaching hospital.

**Population or Sample:**

Women considering surgical management of uterine/vault prolapse or SUI. Exclusion criteria included those under 18, unable to communicate in English or not eligible for all surgical options discussed in the PtDA's.

**Methods:**

A qualitative, semi‐structured interview evaluating women's opinions of the decision aid and the way in which they utilised the PtDA was conducted. The interviews were recorded and transcribed prior to undertaking thematic analysis utilising NVivo software.

**Main Outcome Measure:**

The outcomes of interest were feedback for content, language, format and usage of the PtDA's and women's usage of PtDA's in decision‐making.

**Results:**

Amendments suggested included removal of mesh from the SUI PtDA as this is not routinely available on the NHS and addition of a statement regarding the use of mesh in prolapse surgery. Additional anatomical diagrams were suggested. The need for a robust and regular update system was highlighted as was the provision of foreign language, audio and electronic versions.

**Conclusions:**

PtDA's need updating to ensure relevant content. Clear, detailed and relevant information is required alongside active clinician engagement to reach a mutually agreeable treatment plan.

## 1 Introduction

‘No decision about me without me’ [1] is the headline statement from the United Kingdom Government in response to a move towards greater shared decision‐making and patient autonomy [2]. The General Medical Counsel (GMC) state; ‘All patients have the right to be involved in decisions about their treatment and care and be supported to make informed decisions if they are able’ [3].

Clinicians have a duty to ensure that all information is provided to patients in an accessible, understandable and non‐biased manner. This allows patients to make a fully informed decision about their treatment options, accounting for individual preferences and circumstances. Patient decision aids (PtDAs) have been developed in numerous areas across medicine and surgery to facilitate this.

In situations where there is no definitive best treatment option, individual preferences and values necessarily inform the final decision. PtDAs function by providing all the information required to facilitate shared decision‐making between patient and clinician and aiding in establishing the most important aspects relating to treatment for that individual patient [4].

Informed consent is central to the practice of medicine and in the Cumberlege report, ‘First do no Harm’ (https://www.immdsreview.org.uk/Report.html) it was highlighted that a lack of informed consent led to many of the problems relating to the use of vaginal mesh. In order to standardise the process of consent the National Institute for Clinical Excellence (NICE) in the United Kingdom has developed and published three PtDAs for women who are eligible for pelvic floor surgery. The decision aids are entitled: ‘Surgery for Uterine Prolapse’ [5] ‘Surgery for Vaginal Vault Prolapse [6]’; and ‘Surgery for Stress Urinary Incontinence [6]’ and were published in April 2019.

This study aims to evaluate the usability, acceptability and utility of these three PtDAs as part of routine clinical care. To the best of our knowledge, this is the first published study evaluating the PtDA's as part of routine clinical care. The primary outcome is women's thoughts and opinions regarding the content, layout, language, format and usage of each individual PtDA. The secondary outcome is the evaluation of the broader themes and discourse around decision‐making in a clinical context.

## 2 Methods

Women attending the Urogynaecology outpatient department or outpatient urodynamics at a tertiary NHS teaching hospital who were eligible for pelvic floor surgery for uterine prolapse, vault prolapse or stress urinary incontinence were invited to participate in the study. Participants were given a paper copy of the relevant patient decision aid by the clinician (R.A., R.B. or S.J.) [5, 6, 7] in addition to receiving routine clinical counselling and patient information leaflets at the clinician's discretion.

Inclusion criteria included the ability to communicate in English, aged over 18 and being able to give informed consent. Women who were not eligible for all procedures listed in the relevant PtDA were excluded from the study.

The PtDA was designed for a reading age of 11–14 years and was meant to be explained by a healthcare professional. The reading age of all women included in the study was above this.

This study had a qualitative and a quantitative component to it. A sample size calculation was undertaken for the quantitative component of the study, and 35 women were recruited to each of the three PtDA groups, of whom 10 were invited to each of the PtDA cohorts, giving a total of 30 participants for the study reported. The patient population was mixed, with some women having already undergone surgery at the time of the interview, whereas others were still on the waiting list or had opted for non‐surgical management.

Women were then contacted by the clinical researcher (RA) and invited to participate in a semi‐structured interview lasting between 30 and 60 min (Appendix S1). The interviews took place on Microsoft Teams 365 or on the telephone. All interviews were recorded and transcribed utilising Microsoft Teams 365, either directly on the computer or utilising the telephone on speakerphone with patient consent. The recordings were then reviewed, and the transcriptions edited to reflect the interview verbatim. All recordings were anonymised and stored on a secure NHS drive.

A six‐phase inductive reflexive thematic analysis was undertaken to explore overarching themes around decision‐making and patient choice [8, 9, 10]. Data familiarisation and inductive coding initially generated a broad spread of points of analytical interest. Exploring the data set, we moved between a more deductive framework approach based on Standards for UNiversal Decision Aid Evaluations (SUNDAE) guidelines [11] to allow for exploration and feedback related to the components of the individual PtDA's and an inductive approach, allowing us to examine themes related to decision‐making in more detail across the whole cohort.

All interviews were transcribed and coded by one researcher (RA) and a second researcher (RB) independently coded two randomly selected interviews and the results were discussed between RA, RB and GJ. This collaborative approach was used to enhance understanding, interpretation and reflexivity in our approach to the data.

This mixed method analysis allowed us greater freedom and flexibility to extract both meaningful and practical feedback for the individual PtDA's as well as allowing for reflexive, recursive analysis to generate latent themes surrounding the deeper arguments around patient choice and decision‐making.

Results and analysis are reported in line with the SUNDAE guidelines for evaluating PtDAs [12], focusing on (i) How much and which components were used, (ii) the degree to which it was delivered and used as intended (‘fidelity’).

## 3 Results

The basic demographics of participants for each cohort were reported (Table 1). The mean age for the cohort receiving the uterine PtDA was 63 (range 44–81); 69.5 years for the vault PtDA (range 60–77 years) and 52.1 years for the SUI PtDA (range 39–77 years). The majority of participants were White British (27/30) with two ‘Not stated’ and one ‘White – Other’. Participants were at varying stages through their treatment pathway with 14/30 currently on the waiting list for a surgical procedure; 8/30 having already had a surgical procedure and 8/30 having opted for non‐surgical management.

**TABLE 1:** Patient demographics.

	Vault	Uterine	SUI
Mean age	69.5 (range 60–77)	63 (range 44–81)	52.1 (range 39–77)
Ethnicity	White British (9/10)	White British (9/10)	White British (9/10)
	Not stated (1/10)	Not stated (1/10)	White any other (1/10)
Procedural status at time of interview	Pre‐ procedure (6/10)	Pre‐ procedure (4/10)	Pre‐ procedure (4/10)
	Post‐procedure (3/10)	Post‐procedure (5/10)	Post‐procedure (0/10)
	Conservative (1/10)	Conservative (1/10)	Conservative (2/10)
			Pre‐bulking agent (2/10)
			Post bulking agent (1/10)
			Post Botox (1/10)

### 3.1 Patient Feedback for Individual Patient Decision Aids

The interviews were coded and patient feedback regarding the individual components, delivery and use of the decision aids was collated under the following categories: content, language, accessibility, format, layout and usage (Tables 2, 3, 4).

**TABLE 2:** Feedback for vault patient decision aid.

Feedback: vault	Exemplar quote	No. people
Content		
Addition of table at the end where you can document your own pros and cons for each operation	‘…you put your own pros and cons which I did on another sheet of paper so I can…see which was best for me’	1
Additional anatomical diagrams	‘…the thing that I missed from the decision aid with, but I did get in the leaflet was the diagram of exact bits of anatomy that was about to be operated on…’	3
No recommended amendments	‘…I think it's good as it is, I do honestly’	6
More information about mesh	‘…I don't think it made enough emphasis on that mesh…’	2
More information about recovery	‘…perhaps just a little bit more about recovery time…’	3
Statistical information of use	‘…the percentages and that persuaded me…that's a very good part of it…’	5
Comparison chart was useful	‘…brilliant with the tables and comparisons…’	2
Language		
Language is clear to understand	‘…it explains in words that you can understand’	3
Accessibility		
Foreign language and audio versions	‘…I'm assuming that foreign language versions are available and audio versions are…’ *Note foreign language and audio versions not currently available*	3
Dyslexia and learning disabilities	‘…somebody who's you know things like dyslexia or learning difficulties would find that really hard…’	1
Format		
Electronic format may be of use	‘…I also think it would have been good to have it emailed…’	1
Hard copy preferred	‘…I like that part, it was actually a tactile thing…’	3
Would have read if posted	‘…Yes definitely would…’	9
Would not have read if posted	‘…probably not…’	1
Layout		
Coloured background and lack of graphical contrast	‘…it's quite large colour background to read from…’	1
Clear, simple layout	‘…very well set out…’	2
Usage		
Did not write in PDA	‘…I didn't write in it at all…’	4
Wrote in PDA	‘…I wrote a few notes…’	6

**TABLE 3:** Feedback for uterine patient decision aid.

Feedback: uterine	Quote	No. patients
Content		
Additional anatomical diagrams needed	‘…the woman's anatomy, how it should be, maybe one of the prolapse so people can see what's gone wrong…’	1
Comparison chart was useful	‘…good comparison chart…’	2
Graphical representation of percentage risks was useful	‘…I like the little people symbols you know…good for people to see things…’	4
Good information	‘…I felt all the information was there…’	10
More information about risk	‘…millions of women have had this done surely and yet the data's limited…’	1
More information about recovery	‘…the questions for me were more along the lines of how long it would take to recover…’	3
More information about mesh	‘…I mean mesh has gone out of vogue for lots of reasons hasn't it, but sometimes they need to put it in…’	2
More information about sexual intercourse	‘…maybe a little bit more information on the sex side…’	1
More information about anaesthetic options	‘…anaesthetically as well, do I have a choice?’	1
No recommended amendments to PDA	‘…I wouldn't change anything…’	7
Language		
Doesn't explain what NICE is	‘…it mentions NICE a few times but it doesn't actually say what it is…’	1
Helps break down terminology barriers	‘…it helped you with the appropriate wording…’	1
Language is clear to understand	‘…really clear to read…’	4
Accessibility		
Length of document	‘…some people are put off by a lengthy document…’	2
No barriers to use	‘…I don't think there was anything that put me off using it…’	5
Too much information	‘sometimes you can give people too much information…’	2
Format		
Hard copy of information	‘…actually having something physical for me, yes it's a good thing…’	3
Electronic copy of PDA may be useful	‘…it's *[patient information leaflets]* not the first place they go to to access information’	1
Would need signposting as to importance if posted	‘…you would probably need to to present it in such a way that it made a patient feel that it was really important to read this…’	2
Would have read if posted	‘…I would have read it….’	7
Would not have read if posted	‘…I preferred to be handed it really…’	2
Layout		
Clear layout	‘…It's set out very well…’	3
Confusing layout	‘…it just went on from this little bit of information to the next and it was quite complex…’	1
Usage		
Did not write in PDA	‘…no I hadn't wrote in it…’	1
Wrote in PDA	‘…oh yes, it's covered in scribbles…’	2

**TABLE 4:** Feedback for stress urinary incontinence patient decision aid.

Feedback: stress urinary incontinence	Quote	No. patients
Content		
Additional anatomical diagrams needed	‘…it would be nice if you had the little, you know pictorial diagram how it's going to look…’	1
Comparison chart was useful	‘…what I like about this is it compares all together…’	1
Addition of Botox	‘…I think that needs to be added about the Botox…’ Note: Botox not used in the context of treatment of SUI, only OAB therefore not included	1
Information about intermittent self‐catheterisation	‘…I think that needs explaining…’	2
Information about urodynamics	‘…I do think that perhaps that part needs explaining as well…’	1
Graphical representation of percentage risks was useful	‘…I like the little things with the people…’	3
Good information	‘…I think it's enough…’	5
Expectation of PDA not met	‘…I felt it was more a really useful information source rather than a decision aid…’	1
More information about recovery	‘…it's just the recovery bit….just lacking a little bit…’	1
Mesh should be removed from PDA	‘…I mean at the moment your leaflet is offering something that isn't available…’	2
No recommended amendments to PDA	‘…I think it's alright to be honest…’	6
Needs to be regularly updated	‘…it's just making sure it's kept relevant and up to date…’	2
Language		
Helps break down terminology barriers	‘…if it's there in front of you, it would assist you in having that conversation…’	1
Language is clear to understand	‘…it was easy to read as well…’	4
Accessibility		
Length of document	‘…it's fairly long winded isn't it…’	6
No barriers to use	‘…no definitely not…’	5
Format		
Hard copy of information	‘…I like the fact it's a paper copy…’	4
Electronic copy of PDA may be useful	‘…an email would be good because you've got it, you've got it all the time haven't you…’	3
Would have read if posted	‘…I would have read it…’	5
Would not have read if posted	‘…I don't think so, no…’	2
Layout		
Change order of procedures	‘…I think if it was written the other way around…like the bulking first and then you know probably Botox….then you know your surgery…’	1
Clear layout	‘…really easy to follow…’	2
Usage		
Did not write in PDA	‘…I didn't write in it…’	1
Wrote in PDA	‘…I've written my choice…’	4

Feedback was overall positive, with 19/30 stating that no amendments were required and 27/30 recommending routine clinical use. It is unclear why three patients would not recommend this for routine clinical use.

The graphical statistical representation of statistics and the comparison charts of the different operations were felt to be of benefit across all three PtDA's. There were however aspects common to the content of all three PtDA's that patients felt could be improved upon. These included the use of anatomical diagrams demonstrating normal anatomy; the issue particular to the patient concern; and the post‐operative appearance. Patients also noted the need for more information about recovery, as it was felt that this information was lacking overall.

Concerns about mesh were voiced in all three groups, although in different aspects. Patients within the stress urinary incontinence group commented that, given the pause on mesh continence procedures in the United Kingdom currently, this should be removed from the decision aid to keep the PtDA relevant and up to date. Those patients within the uterine prolapse group and vault prolapse group also felt that more information about mesh may be of relevance, with many being under the impression that mesh had been banned for all urogynaecology procedures, including prolapse operations.

Further information was also requested about anaesthetic options and sexual function for the uterine PtDA, reflecting the requirement for PtDAs to meet the holistic needs of the patient for decision‐making. Some women also felt that more information about intermittent self‐catheterisation was required for the stress urinary incontinence PtDA.

Language was overall felt to be clear to understand although patients noted the occasional use of acronyms such as ‘NICE’ (National Institute for Clinical Excellence) which were not explained. Although a preference for a hard copy of the information was expressed across all three cohorts, it was felt that the option for an electronic copy would be of benefit for some women. When asked if women felt they would have read the document if posted, 21/30 felt that they would have done this. Others expressed a preference to be handed it as part of the consultation. The way in which the PtDA was used varied widely with approximately half (12/30) writing in the PtDA as part of the decision‐making process or sharing it with friends and family (15/30).

The PtDAs were generally felt to be quite lengthy, with the booklets consisting of 17 A4 pages for the uterine and stress urinary incontinence PtDA's and 15 A4 pages for the vault PTDA. There were concerns that this may limit accessibility for some patient groups, including those with dyslexia or learning difficulties. The layout was overall felt to be clear, although a suggested amendment for the SUI PtDA included changing the order of procedures from least to most invasive.

Participants presumed that foreign language and audio versions would be available as routine. At the time of writing, this is not currently the case.

### 3.2 Decision‐Making: Thematic Analysis

The transcripts across all three PtDA's were reviewed in the context of the question ‘how do women make a decision’. Data regarding the individual components of the PtDa's was analysed separately for each separate PtDA.

Themes generated from the overall data set included the following: (1) It's the Doctor's Decision; (2) It's the patient's choice; (3) I don't need a Decision Aid; (4) In wider conversation; (5) It's about me as an individual; (6) It is all about the information.

Here we will briefly expand upon each of those themes, touching in more detail on their interpretation in context within the discussion.

1. ‘It's the Doctor's decision’ describes those women who felt that they would prefer more active guidance from the clinician when choosing their treatment. Many of these women were slightly older and accustomed to a paternalistic system of medicine; ‘…I'm not a surgeon or anaesthetist or anything, so usually do as I'm told…’ (*Vault cohort ID 42*). Overall, within this theme a preference was expressed for a decision to be reached through consultation with the clinician, feeling that the clinician was better situated to make that choice than the individual; ‘See a consultant or a doctor that would say, well for your age…we recommend that. You know, that to me is not taking it out of your hands but it's guiding you, you know’ (*Uterine cohort ID 57*).
2. ‘It's the patient's choice’ is a broad theme, encompassing women's thoughts and feelings around using the PtDA's to make their decision. Key elements highlighted were that women felt the PtDA was useful and helped them make fully informed decisions about their treatment pathway. The PtDA was integral to finding out information about treatment options and women felt confident in making a decision that accounted for their individual circumstances without external pressure. The shift towards patient choice as opposed to a more paternalistic view of medicine was commented on; ‘…we live in a culture now where it's patient choice…’ (*SUI cohort ID 47*). There was however the caveat that clinicians could not completely abdicate responsibility; ‘…I think if it's about the patient being more accountable, then there's got to be an accountable statement for why somebody else…is wanting to choose that option…’ (*Uterine cohort ID 62*). Overall, women felt positively over taking ownership of their decisions and treatment pathway; ‘… it's like giving me that chance to make the decision for myself…’ (*Uterine cohort ID 8*); ‘…you know the choice is yours at the end of the day…’ (*SUI cohort ID 17*).
3. ‘I don't need a decision aid’ reflected the voice of women who felt that they as individuals did not need a decision aid to help them make a choice about their treatment. Many cited that they already knew the information and had often already made their decision prior to reading the PtDA. This was sometimes through previous healthcare experiences or having done their own research prior to the appointment. Others felt that it did not help them to come up with questions and that it was not required to support the conversation with the clinician, so felt irrelevant to their needs. Overall, it was recognised that this would not be the case for all women; ‘…for somebody else who'd not done as much reading around…it would be excellent…’ (*Vault cohort ID 74*) and that it was the individual's choice as to whether or not they used the PtDA; ‘…the choice is the patient's own really…’ (*Uterine cohort ID 53*).
4. ‘In wider conversation’ reflects the way in which the PtDA's were used by the individual and helped inform discussions with clinicians, family and friends. There was an emphasis on revisiting information within the PtDA and having the time to process information after the stress of a consultation or examination; ‘…sometimes when you're speaking to a doctor, you don't always take in what they say’. (*Vault cohort ID 44*). Women were equally divided as to whether they discussed their clinical care with friends and families or not. Most felt that the PtDA helped to break down terminology barriers and facilitated constructive discussion with the clinician; ‘…it's alright saying oh you've got a vault prolapse…I don't know what a vault is apart from a ceiling in a church…’ (*Vault cohort ID 42*). The emphasis was very much on utilising the PtDA to help inform the discussion with the clinician rather than replacing it; ‘…so the decision aid…it can't replace that interaction…’ (*SUI cohort ID 37*). The interplay between individual decision‐making and the influence of the media and experiences of friends of family was also noted.
  ‘It's about me as an individual’ demonstrates how individual requirements impact on decision‐making. There is also a focus on the impact of wider policies such as waiting list times and the impact of restrictions on mesh usage on patients. Some women felt that they would have wanted mesh if it was available; ‘obviously the option that I want [TVT] isn't available on the NHS’ (*SUI cohort ID 79*); whilst others were unsure as to why it was still in the PtDA; ‘there were some things in there that had some uncertainties…using mesh because that was in the decision aid that I had. So I remember thinking, oh, I didn't think that was an option. So I think I just felt a bit confused as to why that was in there.’ (*SUI cohort ID 66*). Patients in the uterine and vault prolapse cohorts often expressed a desire to avoid mesh based on negative publicity; ‘I weren't impressed with the mesh, I've heard things about that mesh’ (*Vault cohort ID 42*).
  Prior knowledge due to experience or profession, patient age and expected recovery time all impacted on choice and timing of procedure. Many felt that they had reached the point where surgery was the only option left to them; ‘I can't live like this, it's got to be done’ (*Vault cohort ID 44*) but cited significant delays impacting them; ‘this has been ongoing now…best part of a year and a half…’ (*SUI cohort ID 30*). Some women felt that their age was a significant factor; ‘I also don't want to delay having it because obviously the older you are, the more risky surgery is…’ (*Vault cohort ID 34*) and age was also felt to impact on retention and absorption of information; ‘…it's more difficult to take things in when you get older…’ (*Vault cohort ID 39*).
5. ‘It's all about the information’ describes the PtDAs as comprehensive sources of information; ‘…It explained everything you needed to know….’ (*Vault cohort ID 26*). Women were clear that a strong incentive to read the PtDA was to find out information about their condition and treatment options; ‘I was just desperate to know what it was all about really’. (*Vault cohort ID 60*). There was a clear range of previous understanding with some women having little to no understanding of basic functional anatomy whereas others felt that due to their personal or professional experience they were entering the consultation well informed. Many were unaware that treatments were available; ‘…for me I just thought it were a woman thing you know, from having kids and stuff, something you had to put up with. I didn't realise there were…things you can actually do’. (*SUI cohort ID 50*). The importance of clear, up to date, unbiased and readily accessible information was at the forefront of the conversation.

## 4 Discussion

### 4.1 Main Findings

The main aim of this study was to establish the usability, acceptability and utility of these three PtDAs as part of routine clinical care. We also evaluated broader themes and discourse around decision‐making in a clinical context.

We found that the PtDA's were overall well received and felt to be useful sources of information regarding treatment options. Issues raised included ensuring that the PtDA was available in different languages and formats to ensure accessibility. The requirement for the PtDA's to be kept up to date was highlighted, with concerns raised regarding the inclusion of mesh in the SUI PtDA, given the current pause on the use of mesh for continence procedures. More information regarding mesh was also requested by women in the vault and uterine prolapse groups.

Recommendations for amendments to the PtDAs included the addition of further anatomical diagrams and information about recovery and anaesthetic options.

The impact of the PtDAs on shared decision‐making was felt to be significant, although women's individual preferences regarding the allocation of responsibility for treatment choice between patient and clinician reflected a broad spectrum.

### 4.2 Strengths and Limitations

Strengths of this study include the large number of women interviewed regarding their experiences. This is the first study to our knowledge that has evaluated the use of these PtDA's in routine care. The use of qualitative interviews allowed us to generate rich understandings of the use and acceptability of the PtDA's. Limitations of this study are the homogeneity of the population interviewed, meaning that we are not necessarily able to extrapolate our results for women from different ethnic and cultural backgrounds. Quantitative data regarding the use of the PtDA's was collected alongside this project and will be submitted for publication as a separate article, although we recognise that best practice would be for these results to be reported alongside one another [11]. Results are not directly applicable outside an NHS setting or the United Kingdom where the PtDA are used almost exclusively.

### 4.3 Interpretation (In Light of Other Evidence)

Decision‐making can be viewed as a spectrum, with the paternalistic model as one extreme where the doctor makes the decision on behalf of the patient based on clinical expertise [13]. The other end of the spectrum is referred to as informed decision‐making, where the patient is provided with all the information required to decide of their own accord [14, 15, 16, 17]. Shared decision‐making is in the centre of this spectrum, reflecting the exchange of information and preferences between clinician and patient, allowing for a mutually agreeable decision to be reached [18]. As clinicians, we rarely discuss the nuances between shared decision‐making and informed decision‐making [19] and implementing true shared decision‐making in practice can be a challenge [20].

This reflects the themes generated in our analysis, with women expressing a preference for the clinician to share their own treatment preferences. Although women were overall positive about being able to decide their own treatment pathways, the ongoing requirement for active clinician engagement was highlighted, reflecting the requirement for shared as opposed to informed decision‐making. This is echoed in other studies where women felt that decisions around their treatment ‘required expertise, knowledge and clinical experience that they did not have’ [21, 22].

The 2017 Cochrane review stated that provision of detailed information with a decision aid was felt to improve knowledge of treatment options and enabled women to feel better informed to participate in decision‐making [22]. Shared decision‐making encompasses the influence of friends, family and other healthcare professionals with the patient–clinician dyad not existing solely in isolation [17]. This encompasses our themes ‘Within wider conversation’ where the interaction between environment, media and other individuals was noted as influencing decision‐making and ‘It's all about the information’, reflecting the requirement for appropriate patient information as identified in the Cochrane review [22].

## 5 Conclusion

In conclusion, the contents of the PtDA require regular updating and amending to ensure that they remain relevant in a changing clinical and political context. The inclusion of TVT in the SUI PtDA needs to be reviewed, and a statement on the use of mesh in prolapse surgery needs to be included in the PtDA's for vault and uterine prolapse. The holistic needs of the patient regarding the context in which their decision is made need to be met, including more information regarding recovery, anaesthetic options, impact on sexual function and other aspects such as the requirement for intermittent self‐catheterisation.

The inclusion of anatomical diagrams within the PtDAs would be of benefit for aiding patient understanding of both normal anatomy and the pathology associated with their symptoms. The length of the document is considerable and may present a barrier for usage. The need for alternative formats including foreign language, audio and electronic was noted.

Shared decision‐making requires not only the provision of information to the patient, but also the active participation of the clinician. This requires a more nuanced approach to counselling; however it is essential so that an optimum treatment pathway can be reached. Although not always straightforward to achieve in clinical practice, the views and opinions of the clinician should be shared in a non‐biased manner, accounting for external and personal influences that factor into decision‐making. Further research and training are required into the optimal deployment of patient decision aids in clinical practice.

## Author Contributions

R.A.: Development of project. Acquisition, analysis and interpretation of data. Manuscript authorship. R.B.: Acquisition and analysis of data. Manuscript review and approval. G.J.: Development of the project. Analysis and interpretation of the data. Manuscript review and approval. S.J.: Conception and development of the project. Manuscript review and approval.

## Ethics Statement

IRAS ID 313282, REC 22/PR/0414. IRAS/HRA approval and local ethical approval.

## Conflicts of Interest

The authors declare no conflicts of interest.

## Supporting information


Appendix S1

## Data Availability

The data that supports the findings of this study are available in the Supporting Information of this article.
