# Supplementary material for: Impact of Patients Decision Aids on Shared Decision‐Making and Patient Satisfaction Prior to Pelvic Floor Surgery
Source: BJOG. 2025 Feb 19;132(7):918–26. doi: 10.1111/1471-0528.18103 (PMC12051250; doi:10.1111/1471-0528.18103)
Supplement: Supplementary file 1 — Appendix S1 [file BJO-132-918-s001.docx]

**NICE PDA STUDY – DOES USE OF A PATIENT DECISION AID IMPROVE THE DECISION-MAKING PROCESS FOR PATIENTS CONSIDERING SURGICAL TREATMENT FOR STRESS URINARY INCONTINENCE, UTERINE PROLAPSE OR VAGINAL VAULT PROLAPSE**

**Interview Schedule (evaluation)**

Hello, my name is XX, Clinical Research Fellow in Urogynaecology at Sheffield Teaching Hospitals. Thank you very much for agreeing to talk to me today about your experiences of using the patient decision aid.

If you are happy still, I would like to record the conversation I have with you today. I just wanted to reassure you that anything to say to me will all be anonymised. It will not be possible to identify you on any of the publications arising from this research. The recording will not include your name (just your study ID and initials) and it will be stored on a secure NHS computer in a locked office.

The interview will explore in more depth your thought and experiences around using the patient decision aid. Consequently, this interview may result in the discussion of sensitive, upsetting or embarrassing issues. You can request to stop the interview, stop recording or withdraw from the study at any time.

1. **Do women and staff use this? (Attraction-Does the material appeal to the target audience?)**

- What did you like about the patient decision aid?
- Was there anything that encouraged you to read the patient decision aid?
- Is there anything that encouraged you to read the patient decision aid straight away?
- If you were sent the patient decision aid in the post, would you want to read it to find out more?
- Did you want to read the patient decision aid to find out more about your treatment options?
- Did you use the patient decision aid to help you make a decision about your treatment?

1. **Do women or staff understand the content? (Comprehension -Does the target audience understand the material?)**

- Tell me in your own words what you think the patient decision aid is for?
- Did the patient decision aid help you think about the benefits of different treatment options?
- Did the patient decision aid help you think about the risks of different treatment options?
- How did the patient decision aid help you think about your treatment options?

1. **Does it enable women or staff to make a decision? (Self-efficacy -Does the target audience feel the message is doable/appropriate for them?)**

- Having read the patient decision aid, do you feel able to make a decision?
- Having read the patient decision aid, do you feel confident in the decision you have made?
- Did you feel you were able to make a decision without pressure from others?
- Did you feel you were able to make an informed choice?

1. **Do women or staff perceive it relevant them? (Cultural acceptability -Does the target audience perceive the message to be salient and acceptable?)**

- In what ways do you think the patient decision aid is relevant to women like you?
- Is there anything in the patient decision aid that makes you feel uncomfortable about the content?

1. **Does the message help support the target audience to take action? (Persuasion -Does the message convince the target audience to take action?)**

- In what way did the patient decision aid help you reach a decision about your treatment options?
- How did the patient decision aid help you to talk to a health professional about your treatment options?

1. **Barriers and facilitators to use in practice**

- Would you recommend using this patient decision aid routinely?
- Is there anything that makes you not want to use the patient decision aid?
- What do you think may improve the use of the patient decision aid?
- Do you think anything needs to be added or taken away (not relevant) from the patient decision aid?

1. **Whether or not the women and clinical staff benefit from their delivery**

- How does the patient decision aid differ to the patient information leaflets for the individual procedures?
- How does the patient decision aid compare to a doctor discussing your treatment options in clinic?

1. **How women or staff used the DA**

- Could you describe to me the ways in which you used the booklet?
- Did you look at it once or more than once?
- Did you write in it?
- Did you look at it with anyone else?
- Did you take it with you to clinic?
- Did you talk to a health professional about it?
- Did it help you think of questions relevant to you?

**Summary**

Is there anything else you would to talk to us about regarding your experience of reading the booklet?

Is there anything else you would like to add before we conclude?

Thank you for taking the time to talk to us today.
